# Complex regional pain syndrome following southern pacific rattlesnake (*C. oreganus helleri*) envenoming

**DOI:** 10.1002/ccr3.5019

**Published:** 2021-11-12

**Authors:** Lawrence McLean House, Matthew R Lewin, Ramana K. Naidu, Halil Beqaj

**Affiliations:** ^1^ University of California San Francisco, Pain Management, Anesthesia & Perioperative Care San Francisco California USA; ^2^ Center for Exploration and Travel Health & Ophirex, Inc. California Academy of Sciences San Francisco California USA; ^3^ Marin Health, Medical Director of Pain Management for Marin Health Medical Center Greenbrae California USA; ^4^ Columbia Presbyterian Medical Center New York New York USA

**Keywords:** CRPS, long‐term envenoming syndromes, snakebite

## Abstract

Complex regional pain syndrome (CRPS) has rarely been reported in the setting of snakebite but might be more common than previously reported. We present the third case of CRPS reported in North America and the first resulting from a pit‐viper's bite.

## Graphical abstract

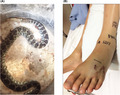

## 1 INTRODUCTION

Long‐term sequelae of snakebite are poorly understood. CRPS Type II following snakebite by a southern Pacific rattlesnake was diagnosed in a 12‐year‐old patient. To our knowledge, this is the first report of CRPS in a pediatric patient and one of the first CRPS Type II by Budapest criteria.

**FIGURE 1:**
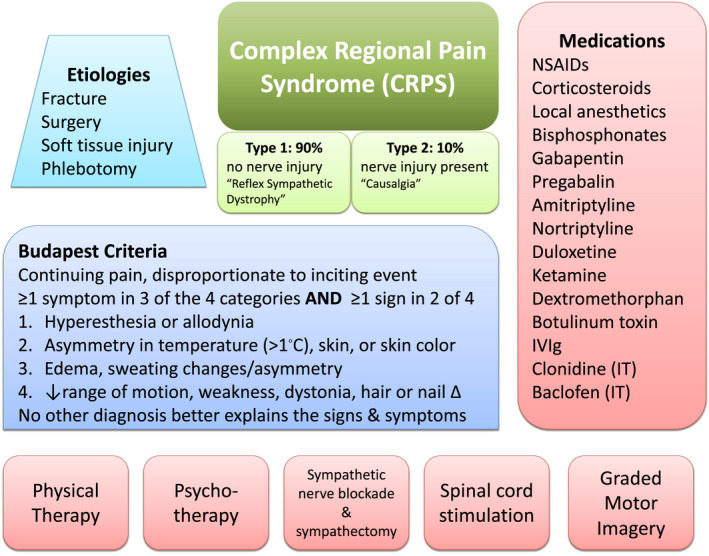
Complex Regional Pain Syndrome (CRPS) has multiple etiologies, including recent identification of snakebite envenoming as a cause. There are many and varied therapeutic approaches including pharmacological, physical and psychological interventions used alone and in combination

Complex Regional Pain Syndrome (CRPS) is known to follow soft tissue injuries and is characterized by persistent pain disproportionate to an initial injury with associated allodynia, autonomic derangement, pseudomotor and skin changes, as well as motor involvement. The sensory disturbances which follow in weeks to months after the injury can be debilitating and require intensive pain management, including physical therapy (Figure 1).

## 2 CASE

**FIGURE 2:**
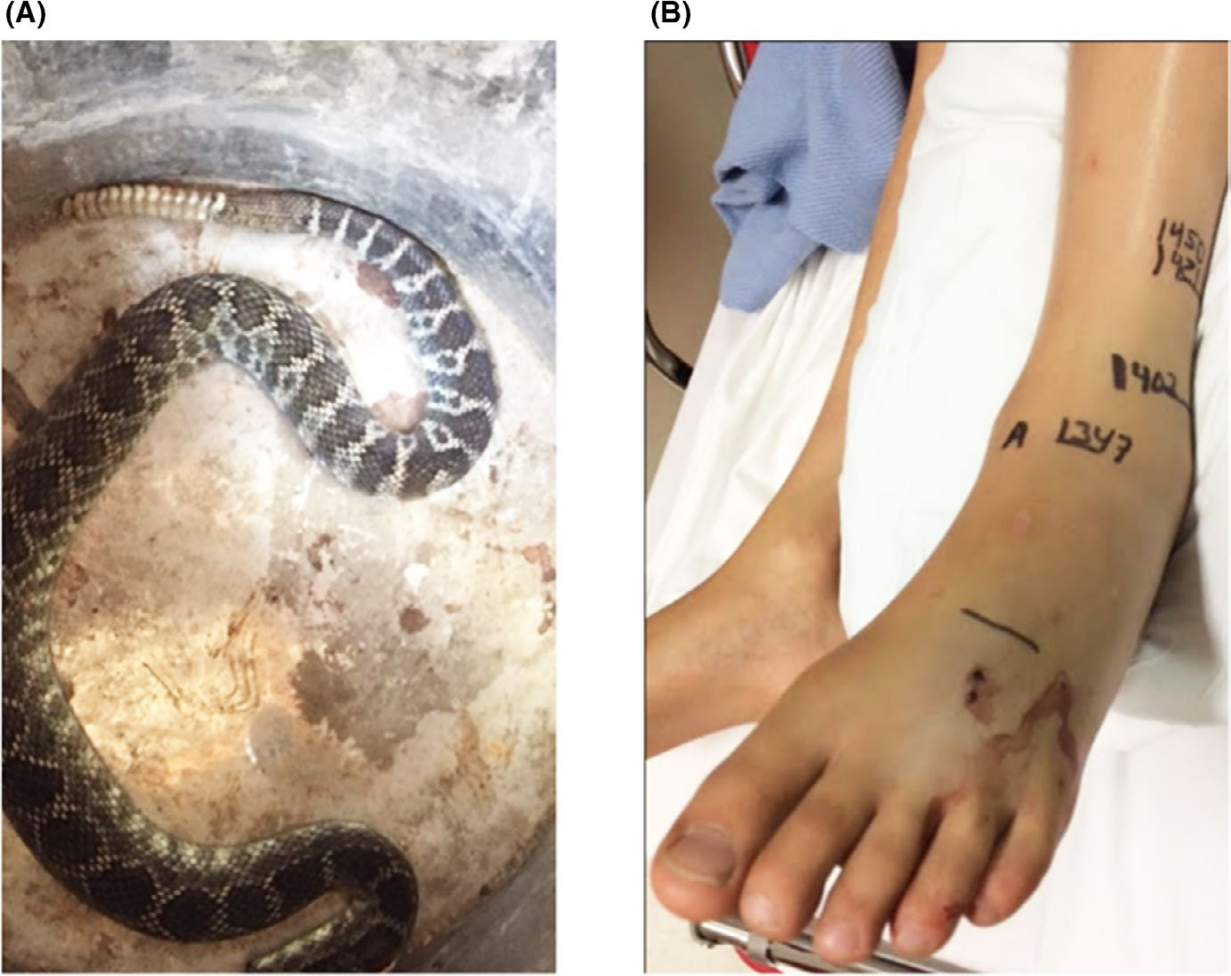
(A) The southern Pacific rattlesnake (*Crotalus oreganus helleri*) that bit the child (B) Fang marks visible on dorsum of the patient's foot and area of bruising in distribution of sural nerve that became symptomatic two months after the incident (photos courtesy of patient's mother)

We present the case of a 12‐year‐old boy presenting with CRPS type 2 appearing two months following snakebite envenoming. Briefly, an unseen southern Pacific rattlesnake (*Crotalus oreganus helleri*) bit him near the lateral malleolus as he walked past a tarp in his uncle's garage in southern California. He described his foot pain from the unknown source as having his foot “lit on fire and run over by a truck.” Search of the garage turned up the snake which was killed and subsequently identified by a local herpetologist (Figure 2). Initial management in the emergency department and subsequent transfer to a tertiary facility comprised intravenous opioids for acute pain management, CroFab^®^ antivenom [Crotalidae polyvalent immune Fab (ovine)], and fluid hydration. Vital signs were within normal limits, and coagulation studies were notable for mildly elevated D‐dimer and fibrinogen levels and mild thrombocytopenia, all of which resolved the next day. Control of progressive envenoming signs was achieved following two additional doses of antivenom totaling 14 vials.

Two months following the initial injury, the patient presented to a chronic pain specialist with progressive symptoms ascending the ipsilateral leg just below the patella in the distribution of the sural nerve. Physical examination was notable for lower extremity edema up to the knee, erythema, hyperalgesia, hot and cold dysesthesia, motor and sudomotor changes in the region overlying the original bite site. These symptoms spread up his leg to the subpatellar area. Budapest Criteria were met for the presence of edema up to the knee, erythema, hyperalgesia, hot and cold dysesthesia, and sudomotor changes.^1^ No further testing such as EMG/NCS was performed nor necessary given the clinical diagnosis. The patient was prescribed physical therapy with a pediatric specialist, which comprised of acupuncture, weight bearing, transcutaneous electrical nerve stimulation, and sensory variation. Active use of the affected extremity was encouraged. Four months after envenoming by the pit viper, the patient was able to ambulate and jog, but only after intensive physical therapy. In the absence of obvious stigmata from the bite, the patient endured skepticism from classmates and teachers he was exaggerating symptoms at school by staying out of physical education activities, ambulating with crutches and keep his leg elevated in class. Four months after the bite and two months after the start of physical therapy, patient was able to ambulate and jog, and had what he felt was a full recovery.

## 3 DISCUSSION

In general, successful management strategies include snake antivenom, gabapentinoids, sympathetic ganglion blockade, physical therapy, graded motor imagery, psychological support and in severe cases, dorsal root ganglion or dorsal column stimulation. At two‐year follow‐up, the patient was symptom free and without persistent physical or psychological sequelae, but this is not necessarily the norm and psychosocial effects of snakebite need to be addressed as well as the immediate effects of acute envenoming.^2^, ^3^, ^4^, ^5^

The financial implications of snakebite and envenoming pose prominent global health concerns. Despite the availability of antivenom treatment in industrialized nations, the cost of this therapy can bankrupt families without medical insurance.^6^ The initial cost of this patient's emergency care totaled more US$70,000, eventually covered by insurance after appeal. These stresses interfere with the psychological wellness of patients as does the uncertainty of recovery time.^6^, ^7^

Progression to CRPS following snake bites and envenoming has only been reported a handful of times in the medical literature, including cases in Nepal, Turkey, Korea, and Norway—all vipers and one each suspected cases induced by an elapid and colubrid of the genera *Micrurus* (coral snake) and *Leptodeira* (cat‐eyed snake), respectively.^8^, ^9^, ^10^, ^11^, ^12^, ^13^, ^14^, ^15^ These disparate geographic locations and two new genera being reported emphasize that venom and/or bites from different genera of snakes can lead to CRPS.^13^, ^15^ In all of the reported cases of CRPS following viper envenoming we found in the literature, symptoms eventually resolved, but often only after significant interventional pain procedures such as stellate ganglion or lumbar sympathetic blocks. In two cases of distal extremity bite injuries, osteoporosis of the involved digits or joints was observed.^8^, ^14^ To our knowledge, the current case is the first described in the Americas in a pediatric patient, a population often more vulnerable to the effects of snakebite envenoming.^16^, ^17^

Snake venom components vary by species, and even intra‐species variation has been observed. Myriad substances within venom lead to inflammation, peripheral sensitization, and local adrenergic receptor upregulation.^18^ Severe bites from *C*. *oreganus helleri* are characterized by mixed neuro‐ and hemotoxic manifestations such as numbness, severe muscle fasciculation, airway compromise and hemorrhage with thrombocytopenia and consumption.^19^ In addition to neurological and hematological manifestations, secretory phospholipases (e.g., crotoxin, a dimeric sPLA2) and zinc metalloproteases lead to endothelial damage and leaky capillaries. Snake venom and hemolysin exacerbates tissue edema by damaging cell membranes and red blood cell lysis. Venoms with high content of sPLA2 such as *C*. *oreganus* species and Mojave (*C*. *scutulatus*) rattlesnakes exhibit both analgesic and nociceptive properties.^20^ In the case of this bite, the toxidrome included severe pain. Whether toxins in venom *per se* induce CRPS via direct nerve injury or by altering physiochemical milieu is very poorly characterized and likely under‐reported.

## 4 CONCLUSION

Emergency medicine providers, primary care physicians and pain physicians should be aware of the potential of snakebites to progress to CRPS. Symptom duration of snakebite and envenoming‐related CRPS can last months to years even with intensive therapy and pain management. Successful management strategies employed in the literature include viper antivenom, gabapentinoids, sympathetic ganglion blockade, physical therapy, and psychological support. Isolation of compounds in viper venoms causing tissue and/or nerve injury may provide insight into the pathogenesis of CRPS. With this case, there are now recorded cases of CRPS from the three groups of venomous snakes found in North America.

## CONFLICT OF INTEREST

MRL receives salary and has shares in Ophirex, Inc. a Public Benefit Corporation.

## AUTHOR CONTRIBUTIONS

HL, RN: Patient care, manuscript, concept; MRL: Patient follow‐up, manuscript, concept; HB: Patient follow‐up, manuscript.

## ETHICAL APPROVAL

This material is the authors’ own original work, which has not been previously published elsewhere. All authors have been personally and actively involved in substantial work leading to the paper, and will take public responsibility for its content.

## CONSENT

Patient and parental consent were obtained and photographs and verification of medical bills provided by the family for purposes of publication.

## Data Availability

Data sharing not applicable to this article as no datasets were generated or analyzed during the current study.

## References

[1] Harden NR , Bruehl S , Perez RSGM , et al. Validation of proposed diagnostic criteria (the ‘budapest Criteria’) for complex regional pain syndrome. Pain. 2010;150(2):268‐274. 10.1016/j.pain.2010.04.030 20493633 PMC2914601

[2] Spano S , Macias F , Snowden B , Vohra R . 237. Snakebite survivors club: ten‐year, retrospective review of crotaline envenomations in central California. Toxicon. 2012;60(2):217. https://doi.org/10.1016/j.toxicon.2012.04.238

[3] Waiddyanatha S , Silva A , Siribaddana S , Isbister GK . Long‐term effects of snake envenoming. Toxins (Basel). 2019;11:193. 10.3390/toxins11040193 PMC6521273 30935096

[4] Williams DJ , Faiz MA , Abela‐Ridder B , et al. Strategy for a globally coordinated response to a priority neglected tropical disease: snakebite envenoming. PLoS Negl Trop Dis. 2019;13(2):e0007059. 30789906 10.1371/journal.pntd.0007059 PMC6383867

[5] Gutiérrez JM , Burnouf T , Harrison RA , et al. A call for incorporating social research in the global struggle against snakebite dimensioning the magnitude and social implications of the snakebite problem. PLOS | Neglected Trop Dis. 2015;9(9):e0003960. 10.1371/journal.pntd.0003960 PMC4574917 26379235

[6] Vaiyapuri S , Vaiyapuri R , Ashokan R , et al. Snakebite and its socio‐economic impact on the rural population of Tamil Nadu, India. PLoS One. 2013;8:e80090. 24278244 10.1371/journal.pone.0080090 PMC3836953

[7] Herzel BJ , Samuel SP , Bulfone TC , Raj CS , Lewin M , Kahn JG . Snakebite: an exploratory cost‐effectiveness analysis of adjunct treatment strategies. Am J Trop Med Hyg. 2018;99(2):404‐412. 29869597 10.4269/ajtmh.17-0922 PMC6090346

[8] Ergan SA , Yoleri O , Yavasi S , Olmez N , Memis A . Complex regional pain syndrome caused by snake bite: a case report. Turk J Phys Med Rehabil. 2012;58:69‐71.

[9] Kleggetveit IP , Skulberg PK , Jørum E . Complex regional pain syndrome following viper‐bite. Scand J Pain. 2016;10(1):15‐18. 10.1016/j.sjpain.2015.07.005 28361765

[10] Seo YH , Park MR , Yoo SH . Development of complex regional pain syndrome after a snake bite: a case report. Korean J Pain. 2014;27(1):68‐71. 10.3344/kjp.2014.27.1.68 24478904 PMC3903804

[11] Ergan SA , Yoleri Ö , Yavaşi S , Ölmez N , Memiş A . Complex regional pain syndrome caused by snake bite: a case report. Turkish J Phys Med Rehabil / Turkiye Fiz Tip Ve Rehabil Derg. 2012;58(1):69‐71.

[12] Thumtecho S , Schimmel J , Trakulsrichai S . Complex regional pain syndrome following a centipede bite: a case report. Clin Toxicol. 2020;58(7):777‐779. 10.1080/15563650.2019.1686515 31771369

[13] Lazaro RP . Complex regional pain syndrome following snakebite: a putatively rare complication of envenomation and review of the literature. Int Med Case Rep J. 2020;13:603. 10.2147/IMCRJ.S275591 33204179 PMC7667582

[14] Bhattarai B , Shrestha BP , Rahman TR , Sharma SK , Tripathi M . Complex regional pain syndrome (CRPS) type‐1 following snake bite: a case report. Nepal Med Coll J. 2008;10(4):278‐280. 19558072

[15] Cruz Salcedo EM , Blanco A , Reed J . Complex regional pain syndrome developing after a coral snake bite: a case report. Cureus. 2020;12(8):e9787. 10.7759/cureus.9787 32953303 PMC7491678

[16] Le Geyt J , et al. Paediatric snakebite envenoming: recognition and management of cases. Arch Dis Child. 2021;106(1):14‐19. 10.1136/archdischild-2020-319428 33115713

[17] Pach S , Le Geyt J , Gutiérrez JM , et al. Paediatric snakebite envenoming: the world’s most neglected ‘Neglected Tropical Disease’? Arch Dis Child. 2020;105(12):1135‐1139. 10.1136/archdischild-2020-319417 32998874

[18] Bickler PE . Amplification of snake venom toxicity by endogenous signaling pathways. Toxins (Basel). 2020;12(2):68. 10.3390/toxins12020068. PMID: 31979014; PMCID: PMC7076764. PMC7076764 31979014

[19] Wasserberger J , Ordog G , Merkin TE . Southern pacific rattlesnake bite: a unique clinical challenge. J Emerg Med. 2006;31(3):263‐266. 10.1016/j.jemermed.2005.09.018 16982358

[20] Zambelli VO , Picolo G , Fernandes CAH , Fontes MRM , Cury Y . Secreted phospholipases A2 from animal venoms in pain and analgesia. Toxins. 2017;9(12):406. 10.3390/toxins9120406 PMC5744126 29311537

